# Relationship between infectious agents for vulvovaginitis and skin color

**DOI:** 10.1590/S1516-31802010000600007

**Published:** 2010-12-02

**Authors:** Rosekeila Simoes Nomelini, Ana Paula Borges Carrijo, Sheila Jorge Adad, Altacílio Aparecido Nunes, Eddie Fernando Candido Murta

**Affiliations:** I MD. Professor in the Instituto de Pesquisa em Oncologia (IPON), Discipline of Gynecology and Obstetrics, Universidade Federal do Triângulo Mineiro (UFTM), Uberaba, Minas Gerais, Brazil.; II Medical student, Universidade Federal do Triângulo Mineiro (UFTM), Uberaba, Minas Gerais, Brazil.; III PhD. Professor in the Discipline of Special Pathology, Universidade Federal do Triângulo Mineiro (UFTM), Uberaba, Minas Gerais, Brazil.; IV MD. Professor in the Department of Social Medicine, Faculdade de Medicina de Ribeirão Preto, Universidade de São Paulo (FMRP/USP), Ribeirão Preto, Brazil.; V MD, PhD. Professor in the Instituto de Pesquisa em Oncologia (IPON), Discipline of Gynecology and Obstetrics, Universidade Federal do Triângulo Mineiro (UFTM), Uberaba, Minas Gerais, Brazil.

**Keywords:** Continental population groups, Vulvovaginitis, Vaginosis, bacterial, Candidiasis, Lactobacillus, Grupos de populações continentais, Vulvovaginite, Vaginose bacteriana, Candidíase, Lactobacillus

## Abstract

**CONTEXT AND Objective:**

Many factors influence occurrences of vulvovaginitis. The aims here were to assess skin color and age-related differences in the vaginal flora and occurrences of vulvovaginitis.

**DESIGN AND SETTING:**

Cross-sectional study; tertiary referral hospital (Universidade Federal do Triângulo Mineiro, Uberaba).

**METHODS:**

Healthy women who underwent routine outpatient gynecological assessments were assessed for vulvovaginitis and vaginal flora and then divided into whites (n = 13,881) and nonwhites (n = 5,295). Statistical analysis was performed using the X^2^ test, logistic regression and odds ratios.

**RESULTS:**

The vaginal microflora was skin-color dependent, with greater occurrence of clue cells, *Trichomonas vaginalis* and coccobacilli in nonwhite women (P < 0.0001). Döderlein bacilli and cytolytic flora were more prevalent in white women (P < 0.0001 and P < 0.05, respectively). The vaginal microflora was age-dependent within the skin color groups. Among the nonwhite women, clue cells were more prevalent in women aged 21 to 50 years; Trichomonas in women up to 40 years and coccobacili in women between 21 and 40 years (P < 0.05). During the proliferative and secretory phases, the nonwhite women were more likely to present clue cells, *Trichomonas, Candida* and coccobacilli (OR, proliferative phase: 1.31, 1.79, 1.6 and 1.25 respectively; secretory phase: 1.31, 2.88, 1.74 and 1.21 respectively), while less likely to present Döderlein flora (OR, proliferative phase: 0.76; secretory phase: 0.66), compared with white women, irrespective of age.

**CONCLUSIONS:**

There are differences in vulvovaginitis occurrence relating to skin color, which may be associated with variations in vaginal flora.

## RESUMO

**CONTEXTO E OBJETIVO:**

Muitos fatores influenciam a ocorrência de vulvovaginites. Os objetivos foram avaliar diferenças relacionadas à cor da pele e idade na flora vaginal e vulvovaginites.

**TIPO DE ESTUDO E LOCAL:**

Estudo transversal; hospital de referência terciário (Universidade Federal do Triângulo Mineiro, Uberaba).

**MÉTODOS:**

Mulheres saudáveis em atendimento de rotina para exames ginecológicos foram divididas em brancas (n = 13.881) e não brancas (n = 5.295) e avaliadas quanto a vulvovaginites e flora vaginal. Para análise estatística, foram utilizados teste X^2^, regressão logística e *odds ratio*.

**RESULTADOS:**

Microflora vaginal foi dependente da cor da pele, com maior ocorrência de “*clue cells*”, *Trichomonas vaginalis* e bacilos cocoides em não brancas (P < 0,0001); bacilos de Döderlein e flora citolítica foram mais prevalentes em brancas (P < 0,0001 e P < 0,05, respectivamente). Flora vaginal foi dependente da idade nos grupos de cor da pele. Entre não brancas, “*clue cells*”, *Trichomonas* e bacilos cocoides foram mais prevalentes nas idades: 21 a 50 anos, até 40 anos, e 21 a 40 anos respectivamente (P < 0,05). Durante as fases proliferativa e secretória, mulheres não brancas tiveram maior probabilidade de apresentar “*clue cells*”, *Trichomonas, Candida* e cocoides (*odds ratio,* OR - fase proliferativa: 1,31; 1,79; 1,6 e 1,25 respectivamente; fase secretória: 1,31; 2,88; 1,74 e 1,21 respectivamente), e menor chance de apresentarem flora Döderlein (OR - fase proliferativa: 0,76; fase secretória: 0,66) comparadas com brancas, independentemente da idade.

**CONCLUSÕES:**

Há diferenças na ocorrência de vulvovaginites relacionadas com a cor da pele, podendo haver associação com variações na flora vaginal.

## INTRODUCTION

Many factors may influence occurrences of vulvovaginitis, including age, vaginal pH, hysterectomy, menstrual cycle phase, HPV (human papillomavirus) infection and skin color. Murta et al. demonstrated, via Papanicolaou staining, that occurrences of infectious agents for vaginitis correlate with age; and further, occurrences of *Candida sp.* in women over 60 years of age are influenced by hysterectomy.^1^ The microflora are interdependent, with a reported association between *Gardnerella vaginalis* and HPV infection.^2^ Moreover, the presence of vaginal flora is affected by the phase of the menstrual cycle, as in the case of cytolytic flora, and by *Candida albicans* abundance,^3^ which in turn impacts local pH. During the proliferative phase of the menstrual cycle and in post-menopausal women, vaginal pH is correlated with endocervical pH.^4^

Racial differences in vaginal flora ecology have been established, and numerous studies have reported differences in occurrences of bacterial vaginosis among racial groups, and specifically, higher prevalence among black women than among white women.^5-7^ However, the majority of these studies evaluated bacterial vaginosis (and other types of vulvovaginitis) solely in pregnant women, which in turn correlated with a greater number of preterm deliveries among black women than among white women.^8^ Bacterial vaginosis, *Trichomonas vaginalis, Chlamydia trachomatis, Mycoplasma hominis* and group B streptococcus colonization were also found to be more frequent in black women, and the presence of one or more of these common microbial conditions was associated with an increased risk of preterm birth.^9^

Bacterial vaginosis and candidiasis are infections relating to alterations in vaginal pH. Several studies have demonstrated that black women have fewer lactobacilli, which maintain vaginal pH via anaerobic metabolism of vaginal glycogen to acidic products.^10^ This is possibly due to overabundance of hydrogen peroxide-producing bacteria, which suppresses the growth of anaerobic bacteria such as lactobacilli.^11,12^ However, the exact cause of this racial difference remains unknown.

## OBJECTIVE

The aim of the present study was to assess potential differences in diagnosing vaginal infectious agents, specifically clue cells and vaginal flora, among non-pregnant white and nonwhite women.

## METHODS

### Participants

A cross-sectional study was conducted at the Cytopathology Service of the Universidade Federal do Triângulo Mineiro (UFTM), Uberaba, Minas Gerais, Brazil, and the Instituto de Pesquisa em Oncologia (IPON), Discipline of Gynecology and Obstetrics (a tertiary referral hospital with free public attendance). The data were collected from the medical records by a single medical student who was participating in a scientific initiation program (Fundação de Amparo à Pesquisa do Estado de Minas Gerais; FAPEMIG).

The presence of clue cells, *Trichomonas vaginalis*, *Candida albicans*, coccoid bacilli, Döderlein bacilli and cytolytic flora was determined via vaginal, cervical and endocervical cytological tests on healthy women who underwent outpatient gynecological assessments at the Discipline of Gynecology and Obstetrics at UFTM between January 1996 and December 2006.

The methodology for collecting cervicovaginal cytological samples is standard in our gynecology service, and the Papanicolaou method was used by the pathologist for the evaluations. During routine pelvic examination on 19,176 non-pregnant women, the uterine cervix was exposed using a sterile and non-lubricated speculum and endocervical samples were obtained by means of a brush, and cervical and vaginal samples by means of a spatula. None of these women were undergoing hormone therapy, or contraception douching or taking vaginal medication. Hysterectomized women were excluded. The cytological smears were fixed in ethyl alcohol and stained using the Papanicolaou method. The samples were evaluated by trained and experienced cytopathologists for the presence of *Trichomonas vaginalis*, *Candida* sp., clue cells, Döderlein bacilli and vaginal flora using the established diagnosis criteria.

After the cytological analysis, the participant pool was divided into two groups according to skin color: (1) white women (described in the records as “white”); and (2) nonwhite women (described in the records as “black” or other mixed skin colors). Information concerning age, pregnancies, parity, smoking and phase of the menstrual cycle was obtained from the participants at the time of sample collection. The health professional who collected the samples described the subjects as white, black or other mixed colors.

For the menstrual cycle analysis, women with cycles of up to 30 days were included (proliferative phase, days 1 to 14; secretory phase, days 15 to 30). Out of the 19,176 patients, 14,442 (75.31%) were included. Among these, there were 6,863 patients (47.52%) in the proliferative phase, of whom 4,987 (72.67%) were white and 1,876 (27.33%) were nonwhite; and 7,579 patients (52.48%) in the secretory phase, of whom 5,619 (74.14%) were white and 1,960 (25.86%) were nonwhite. Each woman was included only once in the series, and it was standardized that only the first Pap smear from each patient would be recorded in the database.

This study was approved by the Research Ethics Committee of UFTM.

### Cytological criteria

The presence of clue cells, *Candida* sp., *Gardnerella vaginalis, Trichomonas,* coccobacilli, Döderlein flora and cytolytic flora was assessed in cell samples that were collected, preserved and stained as described above. Clue cells were defined as squamous cells covered with coccobacilli that presented smudged cytoplasmic borders. *Candida* sp. was identified based upon the presence of pseudohyphae that were weakly stained with eosin or hematoxylin, and/or small spores (diameters of 2-4 mm) that were stained pale pink. *Trichomonas vaginalis* was deemed to be present when an ovoid or round unicellular organism (diameter of 8-20 mm) with pallid or grayish cytoplasm was observed. Eosinophilic granules located within the center of the organism and a vesicular or crescent-shaped nucleus, lightly stained with hematoxylin, were also occasionally observed. Cytolysis was defined as a pale staining; vesicular nuclei with little or no cytoplasm of the intermediate cells predominated in such smears. Coccobacilli were characterized as diffusely scattered bacilli and cocci organisms that occasionally occurred in clumps or microcolonies. Lactobacilli were characterized by the presence of elongated bacillary structures.^13-16^

### Statistical analysis

The results were assessed for statistical significance using the chi-squared test with the Yates correction, multivariate logistic regression and odds ratios, and a 95% confidence interval. The statistical significance level was set at P ≤ 0.05.

## RESULTS

A total of 19,176 participants were evaluated, and they were divided into two groups based on skin color: white women (n = 13,881) and nonwhite women (n = 5,295). The mean age ± standard deviation (SD) was 33.0 ± 9.91 years among the white women, with a range from 13 to 82 years, and 31.1 ± 9.94 years among the nonwhite women, with a range from 12 to 98 years. The smear test was performed on older women because they had symptoms. Table 1 shows the characteristics of each group of patients.

**Table 1.** Characteristics of each group of patients

Epidemiological data	Non-white women n = 5,295	White women n = 13,881
Age, years (mean ± SD)	31.1 ± 9.94	33.0 ± 9.91
Pregnancies (mean ± SD)	2.40 ± 2.34	2.27 ± 2.19
Parity (mean ± SD)	2.17 ± 1.90	2.06 ± 1.69
Smokers	1,519 (28.69%)	3,678 (26.49%)

SD = standard deviation.

The adjusted odds ratio for age, taking into considering the exposure factor of nonwhite skin color is summarized in Table 2. Positive diagnoses of clue cells, *Trichomonas vaginalis* or coccobacilli were more frequent among the nonwhite women (P < 0.0001) than among the white women; while Döderlein and cytolytic flora were more frequent among the white women (P < 0.0001 and P < 0.05, respectively) than among the nonwhite women. The frequency of *Candida* sp. was not skin color-dependent.

**Table 2.** Infectious agents for vaginitis and vaginal microbiota, correlated with skin color

Infectious agents and vaginal flora	Non-white women n %	White women n %	OR (95% CI)	P
**Clue cells**	1,083 (20.45)	2,343 (16.88)	1.27 (1.17-1.37)	**< 0.0001**
***Trichomonas vaginalis***	299 (5.98)	440 (3.17)	1.86 (1.59-2.17)	**< 0.0001**
***Candida sp.***	1,095 (20.68)	2,726 (19.64)	1.07 (0.94-1.11)	0.1109
**Coccoid bacillus**	720 (13.6)	1,563 (11.26)	1.18 (1.09-1.28)	**< 0.0001**
**Doderlein bacilli**	3,399 (64.19)	9,774 (70.41)	0.76 (0.71-0.82)	**< 0.0001**
**Cytolytic flora**	430 (8.12)	1,302 (9.38)	0.83 (0.74-0.94)	**0.0071**

OR = odds ratio; CI = confidence interval.

Yates correlation analysis revealed that skin color and age were significantly correlated in a vaginal flora-dependent manner. There were significant associations for occurrences of clue cells in nonwhite women between 21 and 50 years of age, *Trichomonas vaginalis* in nonwhite women up to 40 years of age and coccobacilli in nonwhite women between 21 and 40 years of age (P < 0.05). Döderlein and cytolytic flora were more prevalent in white women up to 50 years of age and between 31 and 40 years of age, respectively (P < 0.05). The occurrences of *Candida* sp. were not dependent on skin color or age (Table 3).

**Table 3.** Infectious agents for vaginitis and vaginal microbiota, correlated with skin color and age

Infectious agents, vaginal flora and age	Non-white women n %	White women n %	OR (95% CI)	P
**Clue cells**				
≤ 20 years	191 (20.51)	321 (17.95)	1.18 (0.98-1.45)	0.1164
21-30	328 (19.38)	656 (16.37)	1.23 (1.06-1.46)	**0.0067**
31-40	337 (20.99)	765 (17.14)	1.28 (1.11-1.49)	**0.0007**
41-50	208 (21.55)	547 (21.55)	1.36 (1.14-1.64)	**0.0008**
51-60	17 (18.47)	49 (14.80)	1.30 (0.68-2.49)	0.4859
≥ 61	2 (20.00)	2 (7.10)	3.25 (0.27-4.00)	0.5913
***Trichomonas vaginalis***				
≤ 20 years	54 (5.80)	54 (2.64)	1.98 (1.32-2.96)	**0.0006**
21-30	78 (4.60)	106 (3.02)	1.78 (1.31-2.42)	**0.0002**
31-40	117 (7.28)	135 (4.22)	2.52 (1.94-3.28)	< **0.0001**
41-50	46 (4.76)	138 (2.11)	1.13 (0.79-1.62)	0.5280
51-60	4 (4.34)	7 (2.11)	2.10 (0.50-8.23)	0.4121
**Candida albicans**				
≤ 20 years	237 (25.45)	455 (25.44)	1.00 (0.83-1.20)	0.9959
21-30	382 (22.57)	933 (23.28)	0.96 (0.84-1.10)	0.5859
31-40	299 (18.62)	814 (18.23)	1.03 (0.88-1.19)	0.7500
41-50	162 (16.78)	488 (14.95)	1.15 (0.94-1.40)	0.1806
51-60	14 (15.21)	33 (9.96)	1.62 (0.78-3.32)	0.2190
≥ 61	1 (10.00)	2 (7.14)	1.44 (0.10-24.9)	0.6900
**Coccoid bacillus**				
≤ 20 years	163 (17.5)	333 (18.62)	0.93 (0.75-1.15)	0.5075
21-30	349 (20.62)	688 (17.16)	1.25 (1.08-1.45)	**0.0023**
31-40	331 (20.62)	682 (15.28)	1.44 (1.24-1.67)	**< 0.0001**
41-50	187 (19.37)	629 (19.27)	1.01 (0.84-1.21)	0.9778
51-60	24 (26.08)	92 (27.79)	0.92 (0.92-1.59)	0.8472
≥ 61	2 (20.00)	12 (42.85)	0.33 (0.04-2.20)	0.3658
**Döderlein bacilli**				
≤ 20 years	585 (62.83)	1214 (67.89)	0.8 (0.67-0.95)	**0.0092**
21-30	1085 (64.12)	2836 (70.77)	0.74 (0.65-0.83)	**< 0.0001**
31-40	1042 (64.92)	3181 (71.27)	0.75 (0.66-0.84)	**< 0.0001**
41-50	622 (64.45)	2288 (70.00)	0.77 (0.66-0.90)	**0.0010**
51-60	56 (60.86)	212 (64.04)	0.87 (0.56-1.44)	0.6618
≥ 61	8 (8.00)	14 (50.00)	4.0 (0.60-33.00)	0.2018
**Cytolytic flora**				
≤ 20 years	98 (10.52)	197 (11.01)	0.95 (0.73-1.24)	0.7443
21-30	149 (8.80)	390 (9.73)	0.90 (0.73-1.10)	0.2970
31-40	114 (7.10)	433 (9.70)	0.71 (0.57-0.89)	**0.0022**
41-50	60 (6.21)	264 (8.08)	0.75 (0.56-1.02)	0.0642
51-60	9 (9.78)	17 (5.13)	2.00 (0.79-4.97)	0.1627

OR = odds ratio; CI = confidence interval.

During the proliferative phase of the menstrual cycle, the nonwhite women were 1.31, 1.79, 1.6 and 1.25 times more likely to have a positive diagnosis of clue cells, *Trichomonas vaginalis,* *Candida* sp. and coccobacilli, respectively. However, they were 24% and 22% less likely to be diagnosed with Döderlein flora and cytolytic flora, respectively, compared with the white women, regardless of age (Table 4).

**Table 4.** Adjusted odds ratio (OR) for age: an association between non-white women and vulvovaginitis/vaginal microbiota during the proliferative phase of the menstrual cycle

Infectious agents and vaginal flora	OR	CI 95%	P
**Clue cells**	1.31	1.14-1.50	**< 0.0001**
***Trichomonas vaginalis***	1.79	1.37-2.32	**< 0.0001**
***Candida sp.***	1.16	1.37-2.32	**< 0.0001**
**Coccoid bacillus**	1.25	1.09-1.44	**< 0.0001**
**Döderlein bacilli**	0.76	0.67-0.85	**< 0.0001**
**Cytolytic flora**	0.78	0.67-0.85	**< 0.0001**

CI = confidence interval.

During the secretory phase of the menstrual cycle, the nonwhite women were 1.31, 2.88, 1.74 and 1.21 times more likely to have a positive diagnosis of clue cells, *Trichomonas vaginalis,* *Candida* sp. and coccobacilli, respectively. However, they were 34% less likely to be diagnosed with Döderlein flora, compared with the white women, regardless of age. No statistically significant differences were observed among the cytolytic flora during the secretory phase (Table 5).

**Table 5.** Adjusted odds ratio (OR) for age: association between nonwhite women and vulvovaginitis/vaginal microbiota during the secretory phase of the menstrual cycle

Infectious agents and vaginal flora	OR	95% CI	P
**Clue cells**	1.31	1.14-1.49	< 0.0001
***Trichomonas vaginalis***	2.88	2.34-4.05	< 0.0001
***Candida sp.***	1.74	1.62-2.06	< 0.006
**Coccoid bacilli**	1.25	1.09-1.44	< 0.0001
**Döderlein bacilli**	0.66	0.59-0.74	< 0.0001
**Cytolytic flora**	0.92	0.77-1.09	0.34

CI = confidence interval.

## DISCUSSION

The current evidence suggests that occurrences of bacterial vaginosis may be related to race. This includes recent reports of higher frequency of bacterial vaginosis in black women;^17-19^ relationships between bacterial vaginosis, premature delivery^20^ and pelvic inflammatory disease;^21^ and higher risk of preterm birth among black women.^9^ Moreover, genetic factors can influence the susceptibility to infection.^22^ Macones et al. demonstrated that an interaction between genetic susceptibilities (tumor necrosis factor-2 carriers) and environmental factors (bacterial vaginosis) was associated with a higher risk of spontaneous preterm birth.^23^ Ryckman et al. demonstrated that susceptibility to bacterial vaginosis was affected by patterns of genetic variation in stress-related genes, and that smoking played a major role.^24^ Giraldo et al. demonstrated that the incidence of vulvovaginitis differed according to race among Brazilian women, and MBL2 codon 54 gene polymorphism was associated with both recurrent vulvovaginal candidiasis and bacterial vaginosis.^25^

Furthermore, other factors may be involved. A recent study showed a higher number of stressful life events (such as socioeconomic status and psychosocial stress) was significantly associated with higher bacterial vaginosis prevalence, among both African-American and white American women.^26^ Significant differences in cytokine concentrations between women with and without bacterial vaginosis have been demonstrated, and ethnic differences in cytokine concentrations have also been observed in women with normal flora, thus showing that white and black women with normal flora have different cytokine levels, but respond to bacteriosis vaginal in a similar manner.^27^

Elevated vaginal pH, which is prevalent among black women, is also associated with preterm delivery,^28,29^ although racial differences relating to vaginal pH have yet to be definitively identified. Furthermore, black pregnant women are more susceptible to bacterial vaginosis than are white women,^5-7^ thus suggesting a possible link between bacterial vaginosis and race. The relationship between genetic susceptibility and bacterial vaginosis has not been well defined. There are some studies showing this relationship, but others have not seen any correlation.^23,30^ In the present study, we investigated this hypothesis further and determined that occurrences of clue cells, *Trichomonas vaginalis* and coccobacilli were greater in nonwhite women (P < 0.0001) than in white women (non-pregnant). The results reported here are in agreement with previous reports on the prevalence of *Mycoplasma hominis*, *Trichomonas vaginalis*, bacterial vaginosis, group B streptococci, *Neisseria gonorrhoeae* and *Chlamydia trachomatis* among African Americans.^31^ However, it is important to note that additional factors, such as sexual behavior, hormonal status, vaginal devices and abnormal vaginal bleeding may also influence the vaginal microflora constituents.

Alterations to the vaginal microbiota that indirectly affect ovarian function, such as due to bacterial vaginosis and other vaginal infections, are typically associated with changes in pH.^32^ Vaginal pH fluctuations have been observed in premenopausal women during the menstrual cycle^33^ and in postmenopausal women after estrogen administration,^34^ thus suggesting that vaginal pH is related to ovarian hormones. Among premenopausal women, ovarian hormones facilitate vaginal colonization with Döderlein lactobacilli, which convert vaginal glycogen to lactic acid, thereby maintaining vaginal acidity. After the menopause and the accompanying decrease in circulating estrogens, the vaginal pH increases and glycogen and lactobacilli gradually decrease.^33^ Thus, vaginal pH is lower in premenopausal than in postmenopausal women,^4^ and age is strongly associated with an increase in vaginal pH.^35^ These reports suggest that vaginal flora and pH can influence the incidence of vulvovaginitis, and the data reported here suggest that vulvovaginitis is directly associated with ethnicity, since clue cells, *Trichomonas* and coccobacilli were more frequent in nonwhite women. Furthermore, we observed that Döderlein and cytolytic flora were more frequent in white women than in nonwhite women (P < 0.0001 and P < 0.05, respectively), although occurrences of *Candida* sp. were independent of skin color. Hysterectomy can also influence occurrences of vulvovaginitis in relation to changes in the vaginal flora.^1,36^

Among healthy adolescent women who do not have bacterial vaginosis or any other identifiable genital tract infection, the vaginal pH is more alkaline in black women than in white women.^37^ The equilibrium of a healthy vaginal ecosystem is maintained by means of lactic acid production. Lactobacilli exert their growth inhibitory effect on other bacteria via mechanisms involving the production of lactic acid, hydrogen peroxide and bacteriocin.^38^ On the other hand, Fiscella and Klebanoff demonstrated there was no significant differences in vaginal pH levels between black and white women after controlling for confounding factors, particularly vaginal flora.^39^

From analysis on the relationships between skin color and age, we found that there were significant correlations between the presence of clue cells, *Trichomonas vaginalis* and coccobacilli in nonwhite women aged 21-50 years, those aged up to 40 years and women aged 21-40 years, respectively (P < 0.05). Döderlein and cytolytic flora were associated with white women aged up to 50 years and of 31-40 years of age, respectively (P < 0.05). Our patients were of premenopausal age, but it is likely that there is a gradual change in vaginal pH in perimenopausal women, regardless of skin color. Cauci et al. demonstrated that the prevalence of bacterial vaginosis was significantly lower overall in postmenopausal women than in pre and perimenopausal women.^40^ However, many other factors may be involved in this context and may influence occurrences of vulvovaginitis, such as socioeconomic status.^26^

Garcia-Closas et al. demonstrated that the vaginal pH during menstruation (days 1-5) and the proliferative phase of the menstrual cycle tended to be higher than the pH during the secretory phase.^35^ In our study, the nonwhite women were more likely to test positive for *Candida* during the secretory phase of the menstrual cycle than in the proliferative phase (1.74 versus 1.6), which may have been due to the high vaginal pH during the proliferative phase. Furthermore, the nonwhite women were 23% and 34% less likely to test positive for Döderlein flora during the proliferative and secretory phases of the menstrual cycle than were the white women. Moreover, during both menstrual cycle phases, the nonwhite women were more likely to have clue cells, *Trichomonas vaginalis* and coccobacilli. These data strengthen the hypothesis that nonwhite women have fewer lactobacilli, which in turn results in the growth of anaerobic bacteria.^11,12^ In addition, black women remain at a high risk of bacterial vaginosis and intermediate flora (OR 2.2, 95% CI 1.5-3.1), and are more likely to have specific bacterial vaginosis relating to vaginal microflora, gonococcal or chlamydial cervicitis (OR 2.2, 95% CI 1.2-3.8), after adjustment for known bacterial vaginosis risk factors.^41^ From this, it may be possible to ascertain whether acid or neutral pH might contribute towards prophylactic treatment for vaginal infections.

Skin color is difficult for examiners to define, and this may lead to bias. Another limiting factor in this study was that no specific methods for detecting bacterial vaginosis were used, since the study was retrospective. Further research must be conducted in order to investigate the association of vaginal infections with skin color, the relationship between skin color and vaginal flora and the possible contributions of other factors, such as genetic alterations, biological variation and socioeconomic status. Thus, therapies could be used to correct the vaginal pH in nonwhite women as prophylaxis for vaginal infections.

## CONCLUSION

Differences in occurrences of vaginal infectious agents between white and nonwhite women were observed, and these may be associated with changes to the vaginal flora.
